# Does cancer research focus on areas of importance to patients?

**DOI:** 10.3332/ecancer.2016.ed51

**Published:** 2016-01-07

**Authors:** Sing Yu Moorcraft, Amrit Sangha, Clare Peckitt, Rodrigo Sanchez, Martin Lee, Natalie Pattison, Theresa Wiseman

**Affiliations:** 1 The Royal Marsden NHS Foundation Trust, London and Sutton SM2 5PT, UK; 2 Patient representative

## Abstract

The majority of research ideas are proposed by clinicians or scientists and little is currently known about which areas of research patients feel are important. We performed a 4 week pilot patient survey at the Royal Marsden (a specialist cancer centre) to investigate patients’ views on priorities for cancer research. A total of 780 patients completed the survey and the top research priorities were identified as: detection and prevention of cancer, scientific understanding, curative treatment and personalised treatment. The top research priorities were remarkably consistent across age, gender and a variety of tumour types. We believe that patients’ views should be considered alongside those of clinicians and researchers when devising research proposals and strategies.

## Introduction

Tens of billions of dollars have been spent on cancer research worldwide [1]. In 2014, in the United Kingdom (UK) alone, approximately £498 million was spent by the National Cancer Research Institute (NCRI) partners on cancer research [2]. How are decisions made regarding the best way to spend this money and is this truly patient focussed?

Cancer research proposals and strategies are commonly developed by clinicians or scientists and grant applications are reviewed by scientific advisory boards. However, do patients agree with researchers on which areas of research are important?

Although patient involvement in aspects of clinical trials (e.g. the review of trial information) is increasing [3], surprisingly little is known about which areas of research patients feel are important. The main study on this topic is the Macmillan Listening Study, which consulted 105 UK cancer patients and identified 15 areas for research [4]. The top three research priorities identified by this study were: the impact of cancer on patients’ lives/how to live with cancer, risk factors and causes of cancer and early detection and prevention [4].

The Royal Marsden (RM) is a specialist cancer centre based across two sites (London and Sutton) in the UK. In collaboration with the Institute of Cancer Research (ICR), RM is also a Biomedical Research Centre, focussing on cancer research. We conducted the PACER pilot survey at RM to investigate patients’ views on which areas should be priorities for cancer research. Over a 4 week period in April/May 2015, patients were invited to complete an online or paper questionnaire comprising of demographic questions and a list of 12 research themes (with corresponding explanations) to rank in order of perceived priority. Questionnaires were available from reception/stands at a number of locations throughout the hospital and a member of the research team distributed the questionnaire for 2 weeks at each site. In addition, 315 patient members of the RM Foundation Trust were posted or emailed a copy of the questionnaire.

## Results and discussion

We had a total of 780 respondents, of whom 55% were female. The majority of patients were between 46–75 years of age: 119 patients (15%) were less than 45 years, 234 patients (30%) were 46–60 years, 326 (42%) were 61–75 years and 85 (11%) were >75 years. A large number of different tumour types were represented (see Table 1), with the most common being breast, prostate and gynaecological malignancies. Not all patients answered every question, however 396 (51%) patients stated they were being treated with curative intent and 296 (38%) patients were treated with palliative intent. Some patients had not yet started treatment or were having supportive care only, but 397 (51%) of patients were currently undergoing treatment and 229 (29%) were in follow-up/remission.

**Figure 1.**
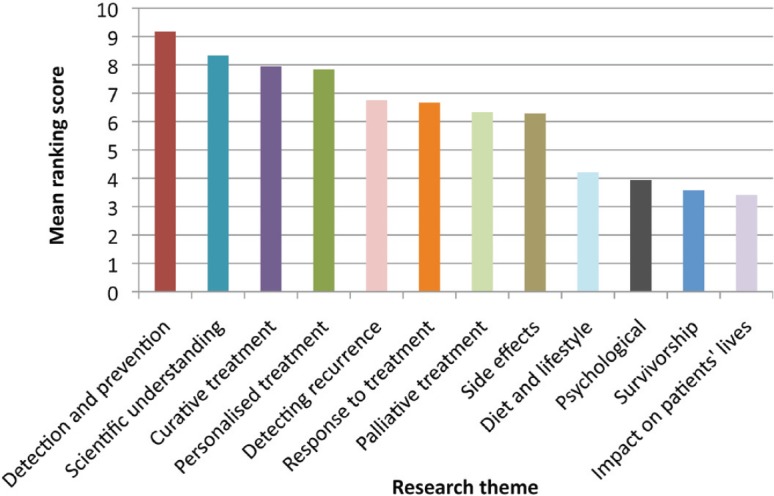
Patients’ ranking of research themes in order of priority.

Patients ranked ‘detection and prevention’ of cancer as their highest research priority, followed by scientific understanding of cancer, curative treatment and personalised treatment (see Figure 1). Gender did not appear to influence patients’ views and the top four research priorities were broadly consistent across all four age groups. However, patients’ ranking of the other research priorities was more influenced by age. Younger patients ranked detection of recurrence and palliative treatment higher than older patients, whereas older patients gave a higher ranking to side effects and response to treatment.

Whether patients were currently receiving treatment or in follow-up did not seem to influence the rankings of the top research priorities, but it was notable that patients who were being treated with palliative intent ranked the palliative treatment option higher than patients who were being treated with curative intent.

The rankings were similar for the majority of tumour types, with the exception of lung cancer, in which curative treatment was ranked the highest priority. However this may have been influenced by the proportion of patients with lung cancer undergoing palliative treatment (72% compared to 21–54% for patients with other tumour types).

We believe that the PACER survey provides an important insight into the views of patients on priorities for cancer research. It is particularly interesting that patients ranked detection and prevention as the highest research priority. This may reflect a sense of altruism as improved strategies for the detection and prevention of cancer will not improve the treatment and prognosis of individual patients who currently have cancer. This is in keeping with previous research, which demonstrated that many patients are motivated to participate in research for altruistic reasons [5, 6].

However, this survey has a number of limitations, especially as it was conducted at a single centre with a mainly white, middle-class population who may have been influenced by the nature of our ongoing research programs. Although we intend to use these results to influence our institutions’ research strategies, we are also planning to conduct a larger, multi-centre survey to investigate if these results are applicable to a wider population.

## Conclusion

Patients’ views are not the only determinants of an optimal research strategy. However, we believe that the views of patients should be considered alongside the views of cancer researchers and clinicians in order to ensure that research strategies are appropriately balanced and patient-focussed. Much cancer research is driven by the pharmaceutical industry, but organisations that provide funding for cancer research (such as charities and government bodies), should take patients’ views into account when reviewing grant applications and developing research strategies. Collaborations with organisations such as the James Lind Alliance can also be helpful in establishing priority-setting partnerships and research strategies [7].

Furthermore, it is important to note that our survey respondents stressed that it was very difficult to rank the research priorities and that if a research theme was allocated a low score, this did not mean that research should not be undertaken in this area. The results of this survey should therefore not be used to exclude certain areas of research from receiving support or funding, but rather to promote research into areas that may be relatively underfunded.

Our survey results also support the move towards allocating more funding for research into early detection and prevention. Historically, this was an underfunded area, accounting for approximately 11% of the NCRI partner spending in 2002, compared to 57% for cancer biology and aetiology and 22% for treatment [2]. In the UK, initiatives such as the National Awareness and Early Diagnosis Initiative (NAEDI) have been established [8] and in 2014 detection and prevention accounted for 20% of the NCRI partners’ research spend [2]. Detecting cancer at an earlier stage has been shown to result in improved outcomes [9], and the greatest gains in survival may result from research in this area, rather than from drugs that result in marginal improvements in survival in the later stages of disease.

## Conflicts of interest

The authors declare that they have no conflict of interest.

## Figures and Tables

**Table 1.** Tumour types of respondents to the PACER survey.

Tumour type	Number of patients (%)
Breast	200 (26%)
Prostate	88 (11%)
Gynaecological	52 (7%)
Colorectal	50 (6%)
Haematological	49 (6%)
Upper gastrointestinal	46 (6%)
Sarcoma	46 (6%)
Lung	46 (6%)
Head and neck	42 (5%)
Renal/urology	40 (5%)
Melanoma	29 (4%)
Other	66 (9%)
‘Don’t know’/not answered	26 (3%)
